# *Clostridium butyricum* Bacteremia Associated with Probiotic Use, Japan

**DOI:** 10.3201/eid3004.231633

**Published:** 2024-04

**Authors:** Ryuichi Minoda Sada, Hiroo Matsuo, Daisuke Motooka, Satoshi Kutsuna, Shigeto Hamaguchi, Go Yamamoto, Akiko Ueda

**Affiliations:** Osaka University Graduate School of Medicine, Osaka, Japan (R.M. Sada, H. Matsuo, S. Kutsuna, S. Hamaguchi, G. Yamamoto);; Osaka University Research Institute for Microbial Diseases, Osaka (D. Motooka);; Osaka University Hospital, Suita, Osaka (A. Ueda)

**Keywords:** *Clostridium butyricum*, bacteremia, probiotics, Japan, inappropriate prescribing, bacteria

## Abstract

*Clostridium butyricum*, a probiotic commonly prescribed in Asia, most notably as MIYA-BM (Miyarisan Pharmaceutical Co., Ltd.; https://www.miyarisan.com), occasionally leads to bacteremia. The prevalence and characteristics of *C. butyricum* bacteremia and its bacteriologic and genetic underpinnings remain unknown. We retrospectively investigated patients admitted to Osaka University Hospital during September 2011–February 2023. Whole-genome sequencing revealed 5 (0.08%) cases of *C. butyricum* bacteremia among 6,576 case-patients who had blood cultures positive for any bacteria. Four patients consumed MIYA-BM, and 1 patient consumed a different *C. butyricum*-containing probiotic. Most patients had compromised immune systems, and common symptoms included fever and abdominal distress. One patient died of nonocclusive mesenteric ischemia. Sequencing results confirmed that all identified *C. butyricum* bacteremia strains were probiotic derivatives. Our findings underscore the risk for bacteremia resulting from probiotic use, especially in hospitalized patients, necessitating judicious prescription practices.

Probiotics have emerged as agents that improve a wide range of conditions and provide essential ingredients for potential health benefits. Probiotics exhibit a diverse array of effects by engaging in competitive interactions with pathogenic microbial communities, competing for binding sites, helping exclude pathogens, and triggering activation of specific genes within and beyond the host’s intestinal tract. This process, in turn, stimulates, regulates, and controls the immune response (*1*). Probiotics have been found to be effective not only in managing conditions such as acute gastroenteritis (*2*) and irritable bowel syndrome (*3*) but also in preventing antibiotic-associated diarrhea (*4*) and even in alleviating symptoms associated with COVID-19 (*5*).

*Clostridium butyricum* is a strictly anaerobic, gram-positive, spore-forming bacillus named for its capacity to produce high amounts of butyric acid. *C. butyricum* was first isolated from the intestines of pigs by Prazmowski in 1880 (*6*), and several strains of *C. butyricum* have been reported from various environments in humans (*7*) and animals (*8*). *C. butyricum* has been detected in the gut of ≈20% of human adults (*9*). Moreover, *C. butyricum* strains were detected in >30% of environmental samples tested (*10*). Some strains of *C. butyricum* are currently used as probiotics and have beneficial effects on humans and animals. One strain of *C. butyricum*, known as *C. butyricum* MIYAIRI 588 (CBM 588), can be found in pharmaceutical probiotics, such as MIYA-BM (Miyarisan Pharmaceutical Co., Ltd., https://www.miyarisan.com), one of the most commonly prescribed probiotics in Japan. CBM 588 has been described as a unique, nongenetically modified strain that does not naturally produce toxins (*11*) or cause disease owing to its susceptibility to the KM1 bacteriophage (*12*). Several confirmatory factors underpin this characterization: it exhibits no propensity for antibiotic resistance transfer, it is devoid of plasmids bearing mobile genetic elements, and it does not possess genes or produce substances related to clostridial toxins, including botulinum neurotoxins A, B, E, and F, or the *Clostridium perfringens* toxins α, β, and ε. Genomic scrutiny of CBM 588 revealed no indicators of pathogenic traits or hemolytic capabilities (*13*). Numerous studies have substantiated the effectiveness of CBM 588, and various animal model experiments have demonstrated its capacity to inhibit the colonization of *Clostridioides difficile* (*14*) and prevent enterohemorrhagic *Escherichia coli* O157 infection (*15*). Human studies have confirmed that CBM 588 prevents antibiotic-associated diarrhea (*16*). In the medical context in Japan, CBM 588 has been prescribed not only for its expected effectiveness as a conventional probiotic but also for the prophylaxis of the diseases we have listed.

There are, however, other strains of *C. butyricum* that are involved in infectious diseases (*17*–*21*). A few case reports have noted the development of *C. butyricum* bacteremia in patients taking probiotics, although strain definition tests using whole-genome sequencing were not conducted (*22*,*23*). Bacteremia caused by *C. butyricum* is a rare condition, and the prevalence, clinical features, and bacteriologic and genetic origins of the strains are unknown. We conducted a single-center, retrospective study of cases of bacteremia caused by *C. butyricum* in Japan to shed light on this clinical event.

## Patients and Methods

### Study Design

We conducted a retrospective cohort study at Osaka University Hospital, a 1,086-bed facility in Osaka, Japan. Our study followed the Strengthening the Reporting of Observational Studies in Epidemiology statement for reporting observational studies (*24*). The Institutional Review Board of Osaka University Hospital approved the study protocol (number 22584(T1)).

### Patients and Baseline Characteristics

To identify cases of *C. butyricum* bacteremia, we reviewed all cases of positive blood culture results for any bacteria that occurred during September 19, 2011–February 5, 2023, from the Laboratory for Clinical Investigation database at Osaka University Hospital. We defined *C. butyricum* bacteremia as cases in which *C. butyricum* was detected in >1 sets of blood cultures. We used MALDI Biotyper (Bruker, https://www.bruker.com/en) to identify *C. butyricum* (*25*). The data we extracted from medical records encompassed such parameters as age; sex; conditions necessitating hospitalization; underlying diseases; placement of a central venous catheter or a peripherally inserted central catheter; presence of polymicrobial bacteremia, including identification of microorganisms other than *C. butyricum*; symptoms at onset; and the updated Charlson Comorbidity Index at the time of bacteremia diagnosis, which was evaluated for every patient (*26*). In addition, for patients who were prescribed MIYA-BM, we checked the MIYA-BM consumption at the point of diagnosis and confirmed the duration of MIYA-BM prescription. We also identified whether MIYA-BM was used for specific reasons in these patients. We defined specific reasons for use of MIYA-BM as treatment for diarrhea, concurrent antibiotic use, or medical history of *C. difficile* infection (CDI), ulcerative colitis, hepatic encephalopathy, or a combination of those conditions. Our investigation involved a detailed evaluation of electronic medical records, which included symptoms of diarrhea occurring ≥3 times/day, concurrent antibiotic use, and medical history of CDI, ulcerative colitis, or hepatic encephalopathy. Finally, we extracted data on the etiology of bacteremia, antibiotic treatment regimens, and mortality within 90 days.

### Microbiologic Information

We determined the MICs for penicillin, ampicillin, cefotaxime, ceftriaxone, cefmetazole, imipenem, meropenem, sulbactam/ampicillin, clavulanic acid/amoxicillin, tazobactam/piperacillin, clindamycin, moxifloxacin, and metronidazole for *C. butyricum* by using the agar dilution method on Brucella agar medium supplemented with 0.5% sheep’s blood. Assays to gauge susceptibility followed the guidelines set by the Clinical Laboratory Standards Institute, tailored for anaerobes (*28*). We assessed the homogeneity of antibiotic susceptibility between the clinical strains and 3 medicinal strains from different lot numbers to evaluate the comparability of their antibiotic susceptibility.

### Whole-Genome Sequencing Analysis

We conducted whole-genome analysis of all strains of *C. butyricum* obtained from blood cultures. In addition, we analyzed *C. butyricum* extracted from MIYA-BM tablets. We then investigated the genetic homology between those strains by evaluating the number of single-nucleotide polymorphisms (SNPs) or insertion/deletion genetic variants between clinical strains and the strain from the MIYA-BM tablets. Finally, we conducted a genomic comparison between clinical isolates of *C. butyricum*, the CBM 588 strain, and other strains of the same species. For the comparison, in addition to the reference strain CDC 51208, we selected 7 strains with fully sequenced genomes that are stored in a bioresource repository.

## Results

We detected 5 blood culture–positive *C. butyricum* bacteremia cases (0.08%) (Table 1) from a total of 6,576 persons who had blood cultures positive for any bacteria (7,484 total clinical strains, including bacteria other than *C. butyricum*). Bacteremia developed in all 5 patients during hospitalization; 3 patients were women and 2 were men. Four patients were immunocompromised: 2 had undergone transplantation, 1 was undergoing chemotherapy for esophageal and gastric cancers, and 1 was receiving multiple immunosuppressive treatments for dermatomyositis. Two of the 5 patients also had end-stage kidney disease and were on dialysis. The Charlson Comorbidity Index scores ranged from 1 to 6 points for each patient. Three patients underwent catheterization with either a central venous catheter or a peripherally inserted central catheter. Four patients were taking prescribed MIYA-BM at the time of bacteremia diagnosis, and 1 patient (no. 2) had been prescribed a different probiotic containing *C. butyricum* 1 month before the diagnosis of bacteremia. All 4 patients taking MIYA-BM were prescribed it >1 week prior to hospitalization, and MIYA-BM was discontinued following the diagnosis of bacteremia in all these patients. Despite a detailed review of the medical records, we were unable to identify the specific reason for prescribing probiotics in 2 patients. All 5 patients had fever and abdominal symptoms, such as diarrhea and pain. One patient (no. 3) with nonocclusive mesenteric ischemia died within 90 days.

**Table 1:** Detailed clinical information on 5 patients with bacteremia caused by *Clostridium butyricum *based on a single-institute, retrospective study, Osaka University Hospital, Japan*

Category	Patient no.				
	1	2	3	4	5
Age, y/sex	68/F	81/F	77/M	53/M	19/F
Onset during hospitalization	Yes	Yes	Yes	Yes	Yes
Diseases requiring hospitalization	Chemotherapy	Immunosuppressive treatment	Post–aortic valve replacement	Simultaneous pancreas and kidney transplant	Double lung transplant
Underlying disease	Esophageal cancer; gastric cancer	Dermatomyositis	Aortic valve regurgitation; end-stage kidney disease	End-stage kidney disease; type 1 diabetes	Idiopathic pulmonary arterial hypertension
Immunosuppression	Yes	Yes	No	Yes	Yes
Charlson Comorbidity Index score	2	1	4	6	1
Central venous catheter insertion	Yes	No	Yes	No	Yes
Concurrent MIYA-BM use	Yes	No, but previously administered another probiotic with *C. butyricum*	Yes	Yes	Yes
Appropriate reason for the prescription of probiotics	Yes (concomitant antibiotic use)	NA	Yes (concomitant antibiotic use)	No	No
Duration of use of probiotics, d	8	NA	12	91	30
Polymicrobial bacteremia, microorganisms other than *C. butyricum*	Yes (MSSA)	Yes (*Enterococcus faecium*/MRCNS)	None	None	None
Symptoms of onset	Fever and diarrhea	Fever and diarrhea	Fever and abdominal pain, septic shock	Fever and abdominal pain	Fever and diarrhea
Diagnosis	Enterocolitis	Enterocolitis	NOMI	Duodenal perforation	Enterocolitis
Antibiotics	CMZ	CTR	MEM	MEM	VCM
90-d mortality	Alive	Alive	Died	Alive	Alive

*CMZ, cefmetazole; CTR, ceftriaxone; MEM, meropenem; MRCNS, methicillin-resistant coagulase-negative staphylococci; MSSA, methicillin-resistant *Staphylococcus aureus*; NA, not applicable; NOMI, nonocclusive mesenteric ischemia; VCM, vancomycin.

A consistent pattern of antibiotic susceptibility was observed in all clinical strains (Table 2). Moreover, those results were consistent with those of previous reports on the antibiotic susceptibility of *C. butyricum*. *C. butyricum* has been reported to be susceptible to penicillin, ampicillin, cefmetazole, imipenem, meropenem, clavulanic acid/amoxicillin, tazobactam/piperacillin, clindamycin, moxifloxacin, and metronidazole but resistant to cefotaxime and ceftriaxone (*11*,*29*,*30*).

**Table 2:** Antimicrobial drug susceptibility of clinical bacterial strains from 5 patients who tested positive for *Clostridium butyricum* in a single-institute, retrospective study, Osaka University Hospital, Japan, and 3 medicinal strains from different lot numbers of *C. butyricum* MIYAIRI 588 strain

Category	Patient strains						Medicinal strains of CBM 588		
Patient no.	1	2	3	4	5				
Strain no.	114–4	129–32	180–11	181–16	216–41		No. 1	No. 2	No. 3
Antimicrobial drug									
Penicillin	0.25	0.25	0.5	0.5	0.25		0.25	0.25	0.25
Ampicillin	0.25	0.25	0.25	0.25	0.25		0.12	0.25	0.25
Cefotaxime	32	32	32	32	32		32	32	32
Ceftriaxone	8	8	16	8	16		8	16	8
Cefmetazole	≤4	≤4	8	≤4	≤4		≤4	≤4	≤4
Imipenem	1	1	2	1	1		1	1	1
Meropenem	≤0.12	≤0.12	0.5	≤0.12	≤0.12		≤0.12	≤0.12	≤0.12
Sulbactam/ampicillin	≤2	≤2	≤2	≤2	≤2		≤2	≤2	≤2
Clavulanic acid/amoxicillin	0.25	0.25	0.5	0.25	0.25		0.12	0.25	0.12
Tazobactam/piperacillin	≤8	≤8	≤8	≤8	≤8		≤8	≤8	≤8
Clindamycin	0.5	0.25	0.5	0.5	0.25		0.25	0.5	0.25
Moxifloxacin	≤0.5	≤0.5	≤0.5	≤0.5	≤0.5		≤0.5	≤0.5	≤0.5
Metronidazole	≤2	≤2	≤2	≤2	≤2		≤2	≤2	≤2

Whole-genome analysis of all 5 patient clinical strains revealed that they either exhibited complete homology or had a maximum divergence of only 19 mutations relative to CBM 588, which was extracted from the MIYA-BM tablets. This result indicates that all clinical strains had the same clone as the CBM 588 extracted from MIYA-BM (Table 3) (*31*–*34*). We performed genetic annotation of the detected mutations (Appendix Table). We performed phylogenetic analysis of *C. butyricum* by using the Type (Strain) Genome Server (*35*). All clinical isolates and probiotics strain were clustered on the same branches (Figure). Average nucleotide identity scores of clinical isolates against those of the probiotics strain were higher than against those of reference strains. This analysis further corroborated the genetic homology between all the clinical strains and the CBM 588 strain.

**Table 3:** Results of whole-genome sequencing of *Clostridium butyricum* obtained from blood culture from 5 patients who tested positive for *Clostridium butyricum* in a single-institute, retrospective study, Osaka University Hospital, Japan

Category	Patient strains				
Patient no.	1	2	3	4	5
Strain no.	114–4	129–32	180–11	181–16	216–41
Average nucleotide identity* against CBM 588 strains	99.986	99.947	99.949	99.943	99.946
All variants†	50	40	63	65	81
Variants not on rRNA region‡	19	1	2	1	0

*Calculated using FastANI (*31*). †Number of all variants in coding genes, which were called and annotated by GATK HaplotypeCaller (*32*) and snpEff (*33*) with annotation information from DFAST (*34*). ‡Number of variants after excluding variants on rRNA region.

**Figure:**
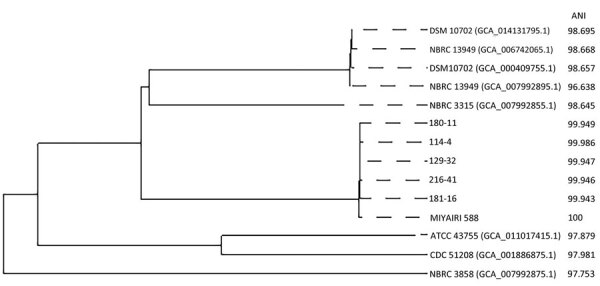
Phylogenetic tree reflecting the relationship between *Clostridium butyricum* MIYAIRI 588, clinical isolates of *C. butyricum*, and 8 reference strains based on data from a single-institute, retrospective study, Osaka University Hospital, Japan. Note: 114-4, 129-32, 180-11, 181-16, and 216-41 represent strain numbers of clinical isolates of *C. butyricum*. MIYAIRI 588 indicates *C. butyricum* MIYAIRI 588. DSM10702 (GCA_014131795.1), NBRC 13949 (GCA_006742065.1), DSM 10702 (GCA_000409755.1), NBRC 13949 (GCA_007992895.1), NBRC 3315 (GCA_007992895.1), ATCC 43755 (GCA_011017415.1), CDC 51208 (GCA_001886875.1), and NBRC 3858 (GCA_007992875.1) represent 8 reference strains. ANI was calculated using FastANI (*31*). ANI, average nucleotide identity.

## Discussion

Our single-center, retrospective study determined that the prevalence of *C. butyricum* bacteremia was 0.08% among all cases with bacteria-positive whole-blood cultures and that all clinical strains were derived from the CBM 588 strain. Bacteremia developed in all patients during hospitalization. Out of 5 cases, 4 had received immunosuppressive treatment and 2 had intra-abdominal issues (1 case of esophageal and gastric cancer and 1 case of post–pancreas and kidney transplantation). 

Ishikawa et al. reported a case series of 11 cases of *C. butyricum* bacteremia, including 3 self-experienced cases and 8 cases from a literature review (*23*). The study revealed that at least 8 cases developed bacteremia during their hospitalization for conditions unrelated to the bacteremia itself. Furthermore, most patients had intra-abdominal issues at the time of developing bacteremia. In 3 cases, *C. butyricum* bacteremia developed after intra-abdominal surgery. Among the 8 cases without intra-abdominal surgery, 6 cases occurred after various intra-abdominal conditions (2 cases of Crohn’s disease, 2 cases of gastrointestinal ulcers, 1 case of biliary tract infection, and 1 case of nonobstructive mesenteric ischemia). Our study results align with previous findings, emphasizing the need for vigilant monitoring of bacteremia development associated with probiotic use in patients with intra-abdominal issues or those undergoing immunosuppressive therapy during their hospitalization.

Our study revealed a high degree of genetic similarity between the strains of *C. butyricum* extracted from MIYA-BM tablets and clinical strains identified through genetic analysis, strongly supporting the definition of probiotic-related bacteremia in all our cases. Reports on probiotic-related bacteremia are scarce. Although systematic reviews of cases of bacteremia after probiotic use have been reported (*36*), to the best of our knowledge, no studies have evaluated the genetic similarities among these reports. Our study offers evidence supporting a direct causal relationship between probiotic prescription and bacteremia. Nonetheless, the patients we identified as nos. 1 and 2 present lingering challenges. We observed 19 differences in terms of SNPs between the strains found in the blood culture of patient 1 and the CBM 588 strain, which was relatively higher than that of the other patients. However, it is common to evaluate strain dissimilarity using fewer than 100 SNPs. Notably, rapidly growing bacteria, such as *Helicobacter pylori*, can accumulate ≈30 SNPs within 6 months of acute infection (*37*). In fact, some studies have established a genetic similarity cutoff of 80 for carbapenem-resistant *Klebsiella pneumoniae* (*38*), suggesting that the genetic dissimilarity observed in this case could be reasonably acceptable. We also considered the possibility that long-term oral administration of probiotics in the past could have led to genetic mutations in the CBM 588 strain within the bodies of patients we examined. Patient 2, who had been prescribed a different probiotic containing *C. butyricum* 1 month before the diagnosis of bacteremia, developed bacteremia caused by the CBM 588 strain. We considered 2 possibilities for this observation: the patient had previously taken MIYA-BM and it had colonized in the patient’s gastrointestinal tract, leading to an infection; or the *C. butyricum* present in the probiotics the patient was taking had genetic similarities to the CBM 588 strain.

Our findings also bring to light the potential adverse effects related to the inappropriate prescribing of probiotics. In all cases where MIYA-BM was prescribed, probiotics were administered for >1 week. However, after a comprehensive review of the detailed medical records, we were unable to identify the appropriate reasons for prescribing probiotics in half of the cases. Probiotics exhibit various therapeutic and preventive effects in different medical conditions, such as averting antibiotic-associated diarrhea (*39*) and CDI (*40*), preventing hepatic encephalopathy in patients with liver cirrhosis (*41*), and managing symptoms in patients with ulcerative colitis (*42*). Although probiotics may demonstrate effectiveness in such specialized clinical scenarios, those scenarios were not observed in the cases we studied, in which probiotics appeared to have been prescribed indiscriminately over an extended period.

One limitation of our study was that it was a single-center, retrospective investigation. Multicenter studies are needed to elucidate the prevalence of *C. butyricum* bacteremia and the genetic origin of the strains. Another limitation was that patient 1 showed improvement with ceftriaxone use, although *C. butyricum* is resistant to it. There is a possibility of contamination resulting from such factors as polymicrobial bacteremia and the absence of central venous catheterization. However, it cannot be ruled out that patients with concurrent sacral pressure ulcers are at risk of developing polymicrobial bacteremia, including *C. butyricum* bacteremia. Also, the duration of probiotic use for each case patient was based on information documented in their medical records, and the precise prescription durations were not always clear. However, the actual prescription periods must exceed the durations documented in the medical records, because the recorded periods represent at least the minimum assessable timeframe. Moreover, although specific reasons for probiotic prescription were not evident in the medical records, unique justifications may have existed. Nevertheless, it is crucial to note that none of the patients had a history of prior antibiotic use, CDI, irritable bowel syndrome, or liver cirrhosis. Hence, the need for prolonged administration exceeding 2 weeks for therapeutic purposes seems unlikely.

In conclusion, our study demonstrates that all clinical strains of *C. butyricum* identified in the positive blood cultures of the 5 cases we analyzed were derived from the strain found in probiotics. Although this type of bacteremia is rare, careful monitoring is essential when bacteremia is caused by probiotics. Clinicians must avoid long-term, inappropriate prescription of probiotics for hospitalized patients with multiple comorbidities, including immunosuppressive treatment and intraabdominal problems, to prevent bacteremia caused by probiotics.

Appendix More information for *Clostridium butyricum* bacteremia associated with probiotic use, Japan.
