# Supplementary material for: Clostridium butyricum Bacteremia Associated with Probiotic Use, Japan
Source: Emerg Infect Dis. 2024 Apr;30(4):665–71. doi: 10.3201/eid3004.231633 (PMC10977840; doi:10.3201/eid3004.231633)
Supplement: Appendix — More information for Clostridium butyricum bacteremia associated with probiotic use, Japan. [file 23-1633-Techapp-s1.pdf]

Article DOI: http://doi.org/10.3201/eid3004.231633

EID cannot ensure accessibility for supplementary materials supplied by authors. Readers who have difficulty accessing supplementary content should contact the authors for assistance.

Single-Center, Retrospective Study Showing *Clostridium butyricum* Bacteremia Associated with Probiotic Use, Japan

Appendix

Appendix Table. Summary of Genetic Mutation Locations and Types of Variants on Genes

strain	variants name	contig name	position	Reference sequence	Alternative sequence	gene_ID	product	Nucleic acid change	Amino acid change	frequency
129–32	129–32_1	contig2	228589	C	T	MGA_3444	hypothetical protein	c.880C>T	p.Arg294*	0.65
180–11	180–11_1	contig2	391259	A	C	MGA_3588	2-hydroxyacid dehydrogenase	c.200A>C	p.Lys67Thr	0.20
	180–11_2	contig3	7	T	TA	MGA_3995	hypothetical protein	c.757dupT	p.Ter253fs	1.00
181–16	181–16_1	contig2	348564	A	T	MGA_3554	AraC family transcriptional regulator	c.801T>A	p.Phe267Leu	1.00
114–4	114–4_01	contig1	113797	C	T	MGA_97	hypothetical protein	c.3865C>T	p.Pro1289Ser	1.00
	114–4_02	contig1	198552	G	A	MGA_154	transposase	c.1432G>A	p.Asp478Asn	1.00
	114–4_04	contig1	427855	A	G	MGA_323	transposase	c.315T>C	p.Asp105Asp	1.00
	114–4_07	contig1	1642093	A	G	MGA_1474	hypothetical protein	c.332A>G	p.Asn111Ser	1.00
	114–4_08	contig1	1660116	G	A	MGA_1489	tryptophan synthase β chain	c.1162G>A	p.Glu388Lys	1.00
	114–4_10	contig1	1819090	T	C	MGA_1625	transposase	c.690T>C	p.Tyr230Tyr	1.00
	114–4_13	contig1	2203803	G	A	MGA_1967	hypothetical protein	c.5G>A	p.Cys2Tyr	1.00
	114–4_14	contig1	2350242	T	C	MGA_2102	hypothetical protein	c.323A>G	p.Glu108Gly	1.00
	114–4_15	contig1	2438117	C	T	MGA_2183	dihydroorotate dehydrogenase B (NAD(+))%2C electron transfer subunit	c.500G>A	p.Gly167Asp	1.00
	114–4_17	contig1	2756750	C	T	MGA_2447	transcriptional regulator	c.217G>A	p.Asp73Asn	1.00
	114–4_18	contig1	3050309	G	A	MGA_2716	hypothetical protein	c.230C>T	p.Thr77Ile	1.00
	114–4_19	contig1	3076183	A	G	MGA_2738	DNA binding response regulator	c.48T>C	p.Ile16Ile	1.00
	114–4_20	contig1	3238758	C	T	MGA_2878	ATPase AAA	c.1405G>A	p.Val469Ile	1.00
	114–4_21	contig1	3269699	G	A	MGA_2905	hypothetical protein	c.2584C>T	p.Pro862Ser	1.00
	114–4_22	contig1	3438483	A	G	MGA_3066	hypothetical protein	c.315A>G	p.Lys105Lys	1.00
	114–4_27	contig1	3512050	A	G	MGA_3130	isoleucine–tRNA ligase	c.1926T>C	p.Phe642Phe	0.99

strain	variants name	contig name	position	Reference sequence	Alternative sequence	gene_ID	product	Nucleic acid change	Amino acid change	frequency
	114–4_28	contig2	333470	T	C	MGA_3541	PTS maltose transporter subunit IIBC	c.343T>C	p.Ser115Pro	1.00
	114–4_32	contig2	724815	C	T	MGA_3944	oxidoreductase	c.553G>A	p.Glu185Lys	1.00
	114–4_33	contig3	106664	G	A	MGA_4088	membrane metallo endopeptidase	c.1316C>T	p.Ser439Leu	1.00
